# Maturation toward neuronal tissue in a Ewing sarcoma of bone after chemotherapy

**DOI:** 10.1186/s13000-016-0516-0

**Published:** 2016-08-09

**Authors:** Maria Carolina Wilhelmina Salet, Rob Vogels, Paul Brons, Bart Schreuder, Uta Flucke

**Affiliations:** 1 Department of Pathology, Radboud University Medical Center, P.O. Box 9101, Nijmegen, 6500 HB The Netherlands; 2 Department of Pediatric Oncology, Radboud University center, P.O. Box 9101, Nijmegen, 6500 HB The Netherlands; 3 Department of Orthopedics, Radboud University Center, P.O. Box 9101, Nijmegen, 6500 HB The Netherlands

**Keywords:** Ewing sarcoma, Pathology, Maturation, Differentiation, Neural

## Abstract

**Background:**

Ewing sarcoma is the second most common bone tumor, occurring mainly in children and young adults. It shows a typical primitive, small round cell morphology and a characteristic fusion oncogene involving *EWSR1* and members of the ETS family in most of the cases. Neuronal maturation after chemotherapy is a rare phenomenon and we herein describe such an exceptional case.

**Case presentation:**

An 8-year old boy was diagnosed with a Ewing sarcoma in the left femur. On biopsy the morphology was typical and there was an *EWSR1-FLI1* gene fusion. He underwent neo-adjuvant chemotherapy and resection of the tumor. On microscopic evaluation, part of the tumor showed ganglioneuroblastoma-like differentiation with expression of neuronal markers. The continued presence of *EWSR1* rearrangement in both the blue round cell component and the ganglioneuroblastoma-like component was shown by FISH analysis.

**Conclusions:**

In conclusion, this case describes the possibility of a Ewing sarcoma to differentiate into a ganglioneuroblastoma-like lesion after neo-adjuvant chemotherapy treatment; the prognostic value of this phenomenon remains questionable.

**Electronic supplementary material:**

The online version of this article (doi:10.1186/s13000-016-0516-0) contains supplementary material, which is available to authorized users.

## Background

Ewing sarcoma, first described by James Ewing in 1921, is the second most common malignant bone tumor after osteosarcoma. Occurring mainly in children and young adults; it accounts for 3 % of childhood malignancies. During the last decades, the 5 year overall survival has improved up to 85 % for patients with localized disease but is still only 30 % for patients with metastatic disease [1, 2]. Ewing sarcomas are primitive small blue round cell tumors of bone and soft tissue, characterized by the expression of *EWS-ETS* fusion genes as a driver mutation [3, 4].

Neural differentiation in Ewing sarcoma is a rare phenomenon and has been described before in a few cases [3, 5]. Here we present a case in which an Ewing sarcoma shows ganglioneuroblastoma-like differentiation after chemotherapeutic treatment.

## Case presentation

An 8-year old boy with no previous relevant medical history, presented with pain and a palpable mass in the left upper thigh. MRI showed a ca. 10 cm large lesion in the femur with soft tissue extension. There were no signs of metastasis elsewhere. The patient received neo-adjuvant chemotherapy by the Ewing 2008 protocol. After 4 cycles of VIDE (vincristine, ifosfamide, doxorubicin and etoposide) chemotherapy, a staging FDG-PET/CT-scan was performed, showing tumor regression, however no normalization of metabolic activity at the primary tumor site. After 6 cycles of VIDE, resection of the femur diaphysis was performed with reconstruction with a vascularised fibula transplant. An optimal recovery has been seen so far, with no sign of recurrence or metastatic disease at 21 months after surgery.

### Histology

#### Biopsy

The pretreatment biopsy (routinely formalin-fixed, paraffin-embedded, decalcified and hematoxylin-eosin-stained) revealed microscopically a small blue round cell tumor with necrosis. A panel of antibodies was applied according to standard protocol (antibodies used can be seen in Additional file 1). There was positive membranous staining for CD99 and CD56, consistent with the diagnosis of Ewing sarcoma. By RT-PCR, the presence of the *EWSR1-FLI1* fusion gene confirmed this diagnosis.

#### Resection specimen

**Fig. 1:**
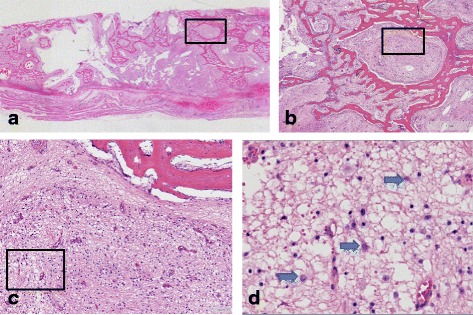
Resection specimen showing ganglioneuroblastoma-like differentiation. **a**: Overview (2.5x) (**b**): 10x (**c**): 20x (**d**): 40x

**Fig. 2:**
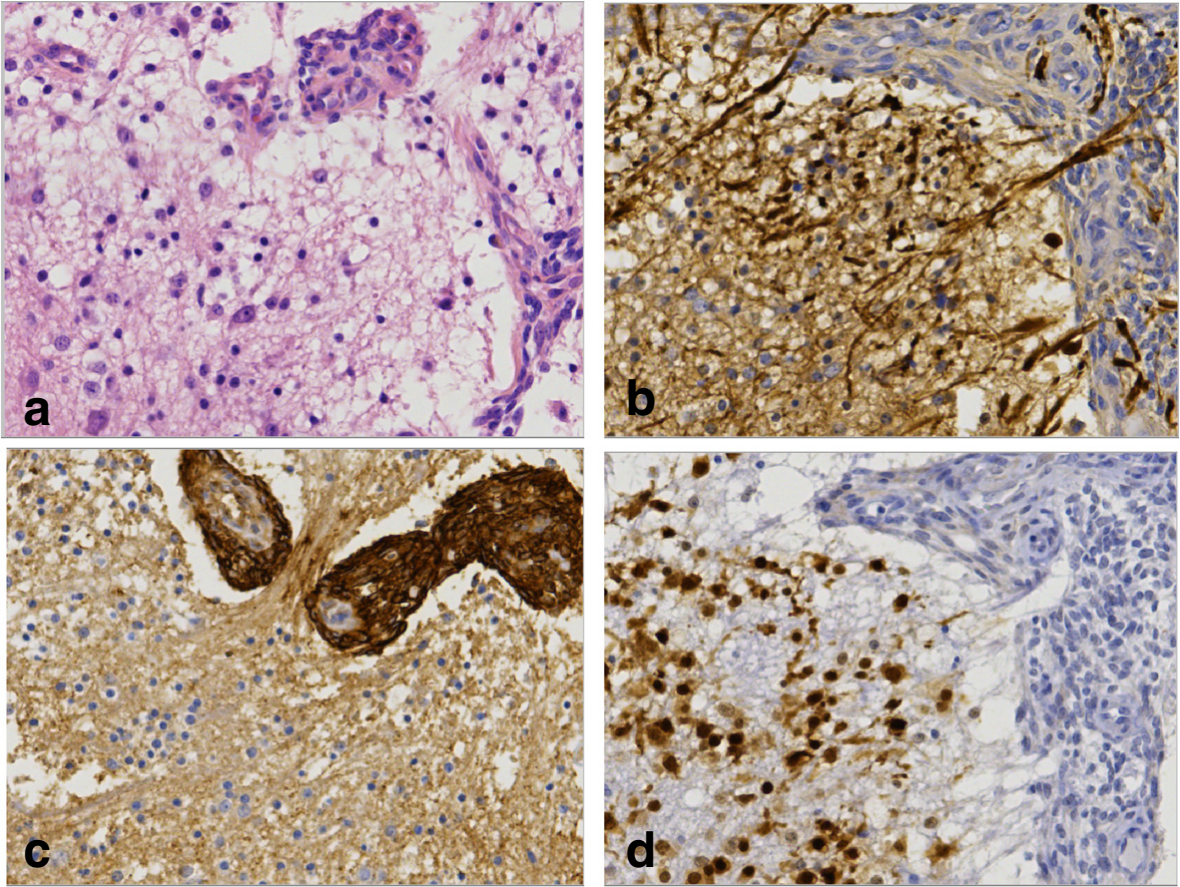
Immunohistochemistry on resection specimen (40x) (**a**) HE showing a part of the tumor with both differentiated (neuronal and ganglion-like cell) and small groups of primitive (typical Ewing) tumor cells (**b**) NF Highlights part of the differentiated tumor cells and the neuropil. The primitive tumor cells are mainly negative (**c**) CD99 labels the primitive tumor cells, whereas the differentiated component is negative. **d** Neu-N shows strong reactivity of the differentiated tumor cells and is negative in the primitive tumor cells

Macroscopically, a 15 cm measuring femur bone and surrounding soft tissue specimen, showed on the cut surface a 10.5 cm large tumor with soft tissue extension. Necrosis, cystic and myxoid changes as well as areas of hemorrhage were present. Microscopy of the tumor showed, beside the usual small blue round cell population, maturated neuroblastoma-like cells and ganglion-like cells against a fibrillary (neuropil-like) background (Fig. 1). It was estimated that of the surviving tumor load about 70 % showed neural differentiation. Again, there was a membrane reactivity for CD99 in the primitive population, whereas the ganglioneuroblastoma-like component showed expression of neural markers, including NF, Neu-N and GFAP. There was no S100 expression (Fig. 2). To confirm the continued presence of the fusion gene FISH analysis was performed showing rearrangement of *EWSR1* in both the small blue round cell component and the ganglioneuroblastoma-like component.

## Discussion

The cellular origin of Ewing sarcomas remains both elusive and controversial. However, the immature neural phenotype of many tumors, formerly known as PNET [5, 6], along with their gene expression signatures and their disposition to neural differentiation in experimental models implicate a neural crest stem cell genetic program as integral feature of pathogenesis of Ewing sarcoma [7]. Therefore, it is not surprising that patient material, at least in some cases, show (ganglio) neuroblastoma-like morphology.

Delattre et al. and Burchill et al. described neuroblastoma-like features in a few untreated Ewing sarcoma cases with genetic confirmation [8, 9], which could be a diagnostic pitfall.

On the other hand, our case and the reported cases by Maeda et al. and Collini et al. show ganglioneuroblastoma-like maturation after chemotherapy probably as treatment effect (3,5).

Whereas Maeda et al. confirmed their diagnosis molecularly only on biopsy material (5), Collini et al. demonstrated the presence of the *EWSR1-FLI1* fusion gene in both pre- and post-treatment specimens, as we did in our case [3].

However, such cases are very rare, we didn’t find another one when reviewing 26 cases from our archives. This is in line with the results of Delattre et al. investigating a series of more than 90 cases with two neuroblastoma-like instances [8].

Maturation during chemotherapeutic treatment is a more common finding in other primitive pediatric tumors such as rhabdomyosarcoma and neuroblastoma [10–13]. Since neural maturation is very rare in Ewing sarcoma, the prognostic value remains unclear in comparison to e.g., embryonal rhabdomyosarcoma where it is associated with favorable outcome [10, 14]. At least the case by Maeda et al. had a unfavorable outcome with metastatic disease showing typical small blue round cell morphology of Ewing sarcoma in contrast to our case with no signs of recurrence or metastases at 21 month after surgery [5].

## Conclusions

In conclusion, this case describes the possibility of a Ewing sarcoma to differentiate into neural tissue after neo-adjuvant chemotherapy treatment, the prognostic value of this phenomenon remains questionable.

## Abbreviations

CT, computed tomography; FDG, fluordeoxyglucose; GFAP, glial fibrillary acidic protein; HE, hematoxylin-eosin; MRI, magnetic resonance imaging; Neu-N, neuronal nuclei; NF, neurofilament; PET/CT, positron emission tomography–computed tomography; PNET, primitive neuroectodermal tumor

## Additional file

Additional file 1: The used antibodies. (XLSX 10kb)
